# Effect of Production Bias on Radiation-Induced Segregation in Ni-Cr Alloys

**DOI:** 10.3390/ma16237475

**Published:** 2023-12-01

**Authors:** Merve Gencturk, Abdurrahman Ozturk, Karim Ahmed

**Affiliations:** 1 Department of Nuclear Engineering, Texas A&M University, College Station, TX 77843, USA; mgencturk@tamu.edu (M.G.); aozturk@tamu.edu (A.O.); 2 Department of Materials Science and Engineering, Texas A&M University, College Station, TX 77843, USA

**Keywords:** Radiation-Induced Segregation (RIS), production bias, radiation damage

## Abstract

We present an in-depth investigation into the Radiation-Induced Segregation (RIS) phenomenon in Ni-Cr alloys. All the pivotal factors affecting RIS such as surface’s absorption efficiency, grain size, production bias, dose rate, temperature, and sink density were systematically studied. Through comprehensive simulations, the individual and collective impacts of these factors were analyzed, enabling a refined understanding of RIS. A notable finding was the significant influence of production bias on point defects’ interactions with grain boundaries/surfaces, thereby playing a crucial role in RIS processes. Production bias alters the neutrality of these interactions, leading to a preferential absorption of one type of point defect by the boundary and consequent establishment of distinct surface-mediated patterns of point defects. These spatial patterns further result in non-monotonic spatial profiles of solute atoms near surfaces/grain boundaries, corroborated by experimental observations. In particular, a positive production bias, signifying a higher production rate of vacancies over interstitials, drives more Cr depletion at the grain boundary. Moreover, a temperature-dependent production bias must be considered to recover the experimentally reported dependence of RIS on temperature. The severity of radiation damage and RIS becomes more pronounced with increased production bias, dose rate, and grain size, while high temperatures or sink density suppress the RIS severity. Model predictions were validated against experimental data, showcasing robust qualitative and quantitative agreements. The findings pave the way for further exploration of these spatial dependencies in subsequent studies, aiming to augment the comprehension and predictability of RIS processes in alloys.

## 1. Introduction

The International Energy Forum report forecasts an 80% surge in global electricity demand by 2050 [1]. With this escalating demand for electricity, the reliance on nuclear energy, an emission-free energy source, must correspondingly increase. In response, there has been a concerted effort to redefine objectives for nuclear power reactors. The emphasis is on enhancing thermal efficiency by increasing operating temperatures and on safety via adopting novel accident tolerant fuels and structural materials. Reactor materials are subjected to extreme conditions, characterized by elevated temperatures, intense stress, and significant radiation flux. Such conditions often lead to the generation, distribution, and interaction of vacancies and interstitials, which in turn drive drastic changes in the chemical composition and microstructure of those materials. Those changes compromise the mechanical integrity of reactor materials and could become a safety concern [2]. Hence, addressing the degradation mechanisms of nuclear materials is pivotal for the ongoing and prospective applications of nuclear energy.

Radiation-induced segregation (RIS) is one of the most critical mechanisms of degradation in irradiated alloys. It is a kinetic (e.g., a non-equilibrium) process driven by the unequal fluxes of solute atoms arriving at the grain boundaries in alloys under irradiation. This stems from the differences in the exchange rates of vacancies and interstitials with the distinct solute atoms of multi-component alloys. Solute atoms that exchange preferably with vacancies will deplete from the grain boundaries, while those that exchange preferably with interstitials will segregate to/be enriched at the grain boundaries. This local variation in the chemistry of the alloy at the grain boundaries renders them susceptible to localized attacks, increasing the overall probability of structural failure of the alloy [3,4]. For example, the depletion of Cr in austenite stainless steels and Ni-Cr-based alloys from the grain boundary is a known contributor to the development of irradiation-assisted stress corrosion cracking, which could shorten the lifetime of nuclear power plants [2,5]. Generally, RIS can lead to embrittlement, corrosion, and phase instability which are detrimental to material performance in nuclear reactors. Hence, a comprehensive understanding of RIS is instrumental in the design and selection of materials capable of withstanding severe nuclear environments.

Ni-Cr-based alloys display high strength, high resistance to swelling, and excellent corrosion resistance at high temperatures [2]. For that reason, they are paramount materials for current and future advanced nuclear reactors. However, RIS modifies the mechanical properties of Ni-Cr alloys significantly [6,7,8,9]. An early study by Robinson and Jenkins [10] showed that the defect geometries in Ni, Ni-8Cr, and Ni-17Cr were similar and dislocation loop formation has a weak dependence on Cr. In contrast, a study by Garner [11] examined Ni-15Cr and pure Ni samples and concluded that adding Cr reduces swelling in Ni and hinders void nucleation. More recent investigations of RIS paid closer attention to the essential factors influencing RIS such as the atomic structure of the interfaces, temperature, and dose rate. Barr et al. [12] studied radiation-induced segregation response in coherent and incoherent twin grain boundaries in a Ni-5Cr alloy. They reported lower Cr concentrations at the incoherent interfaces. Briggs et al. [13] found that the irradiation temperature has a notable effect on the damage profiles in Ni-5Cr and Ni-18Cr alloys, with intermediate temperatures exhibiting the most pronounced Cr depletion. Moreover, non-monotonic Cr profiles, such as the so-called “W” profile, were observed experimentally in Ni-Cr and Fe-Cr-based alloys [14]. While those profiles were initially believed to be transient, they were subsequently demonstrated to be stable even at higher dpa values [14]. Their development was attributed to the interactions of Cr with other solute atoms or the presence of Cr rich phases by the boundary [14]. Various theoretical models [15,16] have been devised to elucidate defect evolution and RIS across differing scales, encompassing rate theory, cluster dynamics (CD), kinetic Monte Carlo (KMC), atomistic kinetic Monte Carlo (AKMC), Density Functional Theory (DFT), Molecular Dynamics (MD), Object kinetic Monte Carlo (OKMC), and phase-field approaches. While atomistic simulations such as DFT, AKMC, and MD, accurately computes defect interactions’ effects on diffusion and clustering, they are limited to short length and time scales. Continuum models such as rate theory, CD, and phase-field can access longer time and length scales and are computationally efficient. However, these coarse-grained models can only account for the interactions between point defects, solute atoms, and interfaces in an effective manner by utilizing net reaction rates and average rate constants. A multiscale approach can be then leveraged to connect these models and overcome their limitations. The coupling between atomistic and continuum models in a direct way (e.g., strong coupling) is still computationally unfeasible. Instead, weak coupling is often employed in which essential energetic and kinetic parameters such as migration and binding energies, defect production rates, and reaction rate constants are calculated using atomistic simulations and their values are passed to the continuum models [12,17,18,19]. We follow this methodology in our investigation here. To advance the current understanding of RIS in Ni-Cr alloys, we carried out a comprehensive investigation of the effects of the main six factors known to influence RIS in alloys, e.g., surface efficiency, grain size, production bias, dose rate, temperature, and sink density. We conducted simulations that test the effect of those factors individually and in different combinations to shed light on the intricate nature of the RIS process. The investigation of separate and coupled effects in a single study and the focus on the implications of production bias on RIS are two unique aspects of our work compared to existing literature. Production bias is often observed under cascade damage conditions of materials irradiated by ions or neutrons. While its effect on void swelling was well documented, using the so-called production bias rate theory model [20,21], and shown to capture the temperature and spatial dependences of swelling, its effect on RIS was largely overlooked. However, our earlier work [22] indicated that production bias triggers the development of surface-mediated patterns of point defects, which may in turn influence the spatial profiles of solute atoms.

The structure of this paper is organized as follows. Section 2 provides an overview of the mathematical modeling of RIS in Ni-Cr alloys. In Section 3, we present and discuss the results obtained from our simulations and relate them to available experimental data. Finally, in Section 4, we draw conclusive remarks summarizing the key findings and implications of our study.

## 2. Methodology

The RIS model used here for Ni-Cr alloys is based upon the advanced RIS treatments introduced in [12,17,18,19], which generalized the classical work of Wiedersich [23]. The works in [17,18] treated the grain boundary as a perfect sink for point defects, keeping the concentration of vacancies and interstitials at the grain boundary at their thermodynamic equilibrium values. This limits the model from explaining the variations in Cr concentration from one boundary structure to another. While the authors in [12,19] also employed a similar model, they pointed out that the sink efficiency for realistic structures of grain boundaries deviates from the equilibrium values. And this deviation was associated with the differences between the predictions of the models and experimental data.

Following the work of [12], we obtained the Ni and Cr vacancy diffusivities from experimental measurements, and the interstitial diffusivities were taken from first-principles-based molecular dynamics simulations (these parameters are summarized in Table 1). The concentrations of vacancies, interstitials, and atoms are determined from mass balance laws taking into consideration that atoms diffuse via vacancy and interstitial mechanisms, which eventually results in strong coupling between the fluxes of atoms and point defects. Specifically, for a binary Ni-Cr system, the balance equations are,

(1) $$\begin{array}{cc} \; & \frac{\partial C_{i}}{\partial t}=-\nabla \cdot J_{i}+K_{0}-K_{iv}C_{i}C_{v}-K_{is}C_{i}C_{s}\\ \; & \frac{\partial C_{v}}{\partial t}=-\nabla \cdot J_{v}+\left(1+b\right)K_{0}-K_{iv}C_{i}C_{v}-K_{vs}C_{i}C_{s}\\ \; & \frac{\partial C_{Cr}}{\partial t}=-\nabla \cdot J_{Cr} \end{array}$$

The time derivatives in Equation (1) represent the evolution of the concentration of interstitials, $C_{i}$, the concentration of vacancies, $C_{v}$, and the concentration of Cr atoms, $C_{Cr}$. On right hand side, $J_{i}$, $J_{v}$, and $J_{Cr}$ are fluxes of interstitials, vacancies and Cr atoms. The second term in Equation (1) is a source term representing defect generation with rate a constant of $K_{0}$. The variable *b* represents the concept of production bias. A positive value for *b* implies a higher concentration of vacancies compared to interstitials. Conversely, a negative value for *b* indicates a higher concentration of interstitials compared to vacancies. The third term, $K_{iv}C_{i}C_{v}$, is a reaction term, referring to the recombination between interstitials and vacancies. The last term is also a reaction term, representing the reaction between the defect *x* (interstitial or vacancy) and the sink *s* with a rate constant of $K_{xs}$.

The diffusivity of element *k* (Cr or Ni) for defect *x*, $d_{x,k}$, is defined by the following equation:

(2) $$\begin{array}{cc} \; & d_{x,k}={d_{0}}^{x,k}\exp\left(\frac{-{E_{m}}^{x,k}}{kT}\right) \end{array}$$

where ${d_{0}}^{x,k}$ is pre-exponential factor of Ni or Cr for vacancy or interstitial, ${E_{m}}^{x,k}$ is the migration energy of Ni or Cr for the vacancy or interstitial, *T* is temperature and *k* is the Boltzmann constant.

We expressed the partial diffusion coefficient by D=dN, where *N* is atomic fraction and *d* is diffusivity (see Equation (2)). The spatial dependence resides in the *N*. In contrast, the *d* is composition independent and contains the kinetic and diffusion information [23].

(3) $$\begin{array}{cccc} D_{i}^{Cr} & =d_{i,Cr}N_{Cr} & D_{i}^{Ni} & =d_{i,Ni}N_{Ni}\\ D_{v}^{Cr} & =d_{v,Cr}N_{Cr} & D_{v}^{Ni} & =d_{v,Ni}N_{Ni}\\ D_{Cr}^{v} & =d_{v,Cr}N_{v} & D_{Cr}^{i} & =d_{i,Cr}N_{i} \end{array}\;$$

where $D_{i}^{Cr}$ and $D_{i}^{Ni}$ are the partial diffusion coefficients for interstitials via Cr and Ni atoms, $D_{v}^{Cr}$ and $D_{v}^{Ni}$ are the partial diffusion coefficients for vacancies via Cr and Ni atoms, $D_{Cr}^{v}$ and $D_{Cr}^{i}$ are the partial diffusion coefficients for Cr atoms via vacancies and interstitials. The total diffusion coefficients for the vacancies, interstitials and Cr atoms are given as $D_{v}$, $D_{i}$ and $D_{Cr}$, respectively.

(4) $$\begin{array}{cc} \; & D_{v}=d_{v,Cr}N_{Cr}+d_{v,Ni}N_{Ni}\\ \; & D_{i}=d_{i,Cr}N_{Cr}+d_{i,Ni}N_{Ni}\\ \; & D_{Cr}=d_{v,Cr}N_{v}+d_{i,Cr}N_{i} \end{array}$$

where $d_{v,Cr}$ and $d_{i,Cr}$ are vacancy and interstitial diffusivities for Cr, $d_{v,Ni}$ and $d_{i,Ni}$ are vacancy and interstitial diffusivities for Ni. Therefore, fluxes take on the following expressions,

(5) $$\begin{array}{cc} \; & J_{v}=d_{v,Cr}\Omega C_{v}\nabla C_{Cr}+d_{v,Ni}\Omega C_{v}\nabla C_{Ni}-D_{v}\nabla C_{v}\\ \; & J_{i}=-d_{i,Cr}\Omega C_{i}\nabla C_{Cr}+d_{i,Ni}\Omega C_{i}\nabla C_{Ni}-D_{i}\nabla C_{i}\\ \; & J_{Cr}=-D_{Cr}\nabla C_{Cr}+d_{v,Cr}\Omega C_{Cr}\nabla C_{v}-d_{i,Cr}\Omega C_{Cr}\nabla C_{i} \end{array}$$

where $J_{v}$ and $J_{i}$ are the fluxes of vacancies and interstitials, $J_{Cr}$ is flux of the Cr atoms. Ω is atomic volume.

We utilized the Multiphysics Object-Oriented Simulation Environment (MOOSE) framework to numerically solve the balance equations of point defects as in our earlier work [22]. Specifically, a fully coupled and fully-implicit scheme was used to obtain numerical solutions under different conditions for this stiff dynamical system. This robust scheme, along with its built-in adaptive time step algorithm, was able to efficiently converge to the steady-state solutions. In our simulations here, we simply solve the balance equations in a Cartesian coordinate system, effectively assuming the grains to be hexahedral. In particular, we conducted 1D simulations using the binary system as a model to gain insights into the phenomenon of RIS in Ni-Cr-based alloys. The properties of Ni and Cr used in our simulations are provided in Table 1.

## 3. Results and Discussion

In our earlier study [22], we investigated the surface and size effects on the behavior of point defects in the presence of production bias. We concluded that those effects are highly sensitive to the value of the production bias. Production bias manifests itself because of the differences in the rates of clustering and the stability of clusters of vacancies and interstitials. This eventually results in unequal number of surviving single point defects at the end of the cascade stage. The value of the production bias, as shown via atomistic modeling and simulations, depends on temperature, crystal structure, composition, and irradiation conditions. Here, we perform a comprehensive study and analysis of the effects of surface, size, production bias, dose rate, temperature, and sink density on the RIS in Ni-Cr. We conducted simulations that test the influence of those factors separately and in different combinations to advance the current understanding of RIS. Moreover, we compare the predictions of our simulations with experimental data whenever possible.

### 3.1. Surface-Mediated Patterns and Size Anomalies

**Figure 1:**
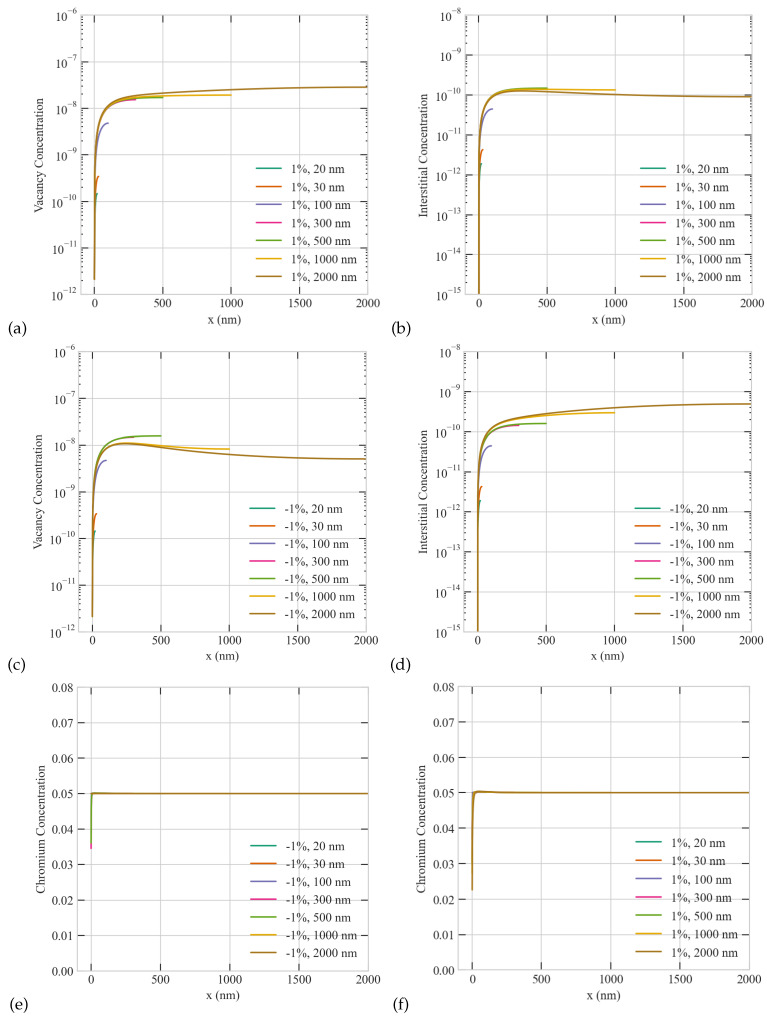
Effect of grain size on the steady-state concentration profiles of point defects and Cr atoms at a temperature of 773 K and dose rate of 5.6 $\times \;10^{-6}$dpa/s for +1% and −1% production bias. Upper row: (**a**) vacancy (**b**) interstitial with positive production bias. Middle row: (**c**) vacancy (**d**) interstitial with negative production bias. Lower row: (**e**) Cr concentrations for the case of negative bias (**f**) Cr concentrations for the case of positive bias.

**Figure 2:**
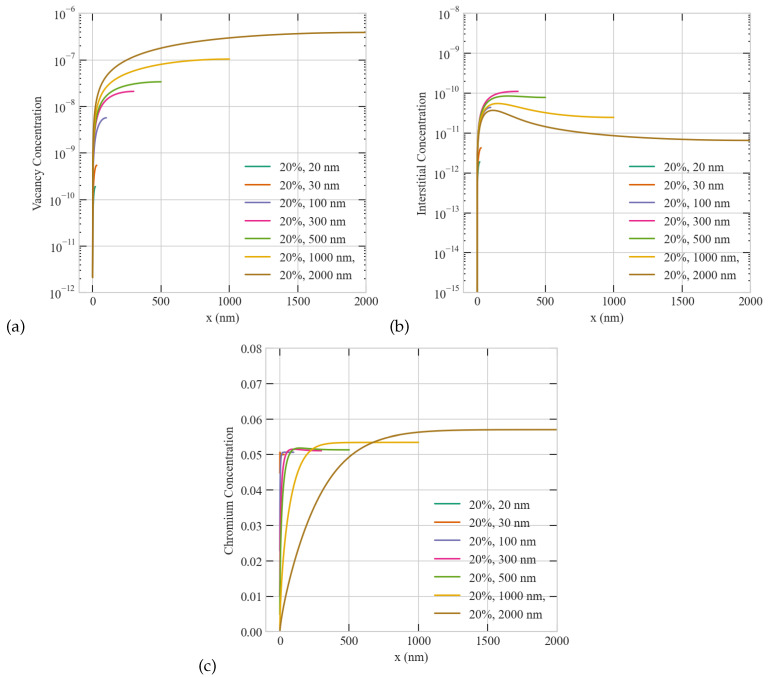
Effect of grain size on the steady-state concentration profiles of point defects and Cr atoms with 20% production bias. Upper row: (**a**) vacancy (**b**) interstitial. Lower row: (**c**) Cr concentrations for 20–2000 nm sizes.

In order to demonstrate the development of surface-mediated patterns and size anomalies in the steady-state concentrations of point defects, we conducted a systematic analysis considering various sizes ranging from 20 to 2000 nm at a temperature of 773 K and a dose rate of 5.6 $\times \;10^{-6}$ dpa/s for three different values of the production bias. A uniform sink density (representing voids/bubbles and dislocations) of 1 $\times \;10^{18}$ m${}^{-3}$ was assumed. Figure 1 and Figure 2 illustrate that, for non-zero production bias, the steady-state profiles are influenced by grain size. For small grain sizes (≤500 nm), the steady-state concentrations of both interstitials and vacancies decrease at the grain boundary (x = 0) and increase towards the center of the grain (at the opposite far end). This reduction in defect concentrations at the grain boundary can be attributed to its role as a sink. This is the classical/expected monotonic behavior of this dynamical system. However, for large grain sizes (>500 nm), the steady-state concentrations of interstitials and vacancies exhibit contrasting trends. Depending on the sign and magnitude of production bias, the steady state profile of one of the species exhibits a peak at the center of the grain while the other species peaks in the vicinity of the grain boundary. The sign of the production bias determines which species displays a maximum at which location while the magnitude affects the value of this maximum point regardless of its location. The exact size at which this transition in the spatial profiles of the steady state concentrations is first noticed is what we call the critical size [22]. At the critical size, the grain’s center and average concentrations of the defect species displaying the non-classical/non-monotonic profile decrease with further increase of size. This reduction in the steady state concentrations with size is what we refer to as size anomaly.

Figure 1 presents those anomalies for the case of low production bias (+/−1%) usually observed under electron or light ion irradiation. For the case of positive bias, vacancies, which are produced at an effectively higher rate than interstitials, accumulate at the center of the grain regardless of size while interstitials peaks close to the grain boundary above the critical size of 500 nm (see Figure 1a,b). The steady state concentration of vacancies at the grain center always increases with size. While the steady-state concentration of interstitials also initially increases with size, it then starts to decrease with size above the critical size. For the case of negative bias, interstitials, which are produced at an effectively higher rate than vacancies, accumulate at the center of the grain regardless of size while vacancies peaks close to the grain boundary above the critical size of 500 nm (see Figure 1c,d). The steady state concentration of interstitials at the grain center always increases with size. While the steady-state concentration of vacancies also initially increases with size, it then starts to decrease with size above the critical size. Hence, the trends in the steady state profiles of vacancies and interstitials are reversed as the sign of production bias is changed, which is consistent with the dynamical system of point defects (recall Equation (1)). These anomalies in the dependence of point defects on size are surface-mediated and triggered by production bias, which breaks the symmetry of the dynamical system. Compared to the case of zero bias, a non-zero bias implies that to reach a steady state the surface/boundary must absorb more of the species that is produced with higher rate. In other words, the surface must be biased towards the species produced at higher rate and against the species produced at lower rate. Therefore, neutral surfaces cannot exist in systems with non-zero production bias. The species absorbed less efficiently by the surface/boundary then tend to accumulate close to that surface/boundary. The resultant patterns and anomalies in the steady state profiles of vacancies and interstitials in turn affect the steady state profile of the Cr solute atoms. As shown in Figure 1e,f, The Cr is depleted more at the grain boundary for the case of positive bias (Figure 1f) than negative bias (Figure 1e). This is because Cr tends to exchange more with vacancies, which are more abundant under the condition of positive bias.

Figure 2 presents those anomalies for the case of high production bias (+20%) usually observed under neutron or heavy ion irradiation. As evident from the figure, the spatial patterns and size anomalies are more apparent at higher production bias compared to the lower production bias conditions (Figure 1). The average steady state concentrations of vacancies/interstitials at +20% bias are higher/lower than their counterparts at +1% bias. Moreover, the critical size is reduced from 500 nm to 300 nm as the production bias increased from +1% to +20%. Furthermore, the Cr depletion becomes more pronounced with bias. The value of Cr at the grain boundary decreases and the depleted region widens as the production bias increases. This is attributed to the higher vacancy to interstitial ratio at +20% compared to +1%. One can predict that those distinct surface-mediated patterns of point defects could in turn influence the formation of extended defects since the prevailing of the presence of one type of point defects in the vicinity of a surface/boundary will facilitate its clustering by that surface/boundary. This in fact has been reported in experiments before. Ma et al. [24] have shown that accounting for production bias was the only plausible explanation for the formation of vacancy loops near the free surface in self-irradiated Ni at 783 K. Specifically, they estimated that at least a production bias of +10% was required to result in the experimentally observed density and size of vacancy loops. Field et al. [25] demonstrated the accumulation of dislocation loops in the vicinity of grain boundaries in the neutron irradiated ferritic FeCrAl. Moreover, Mao et al. [26] conducted a comprehensive experimental study that elucidated the dependence of irradiation tolerance on size and temperature in irradiated Cu and Cu-W alloys. Investigating the connection between the point defect patterns and the resultant non-uniform distribution of extended defects will be the focus of a future work.

### 3.2. Effect of Dose Rate

**Figure 3:**
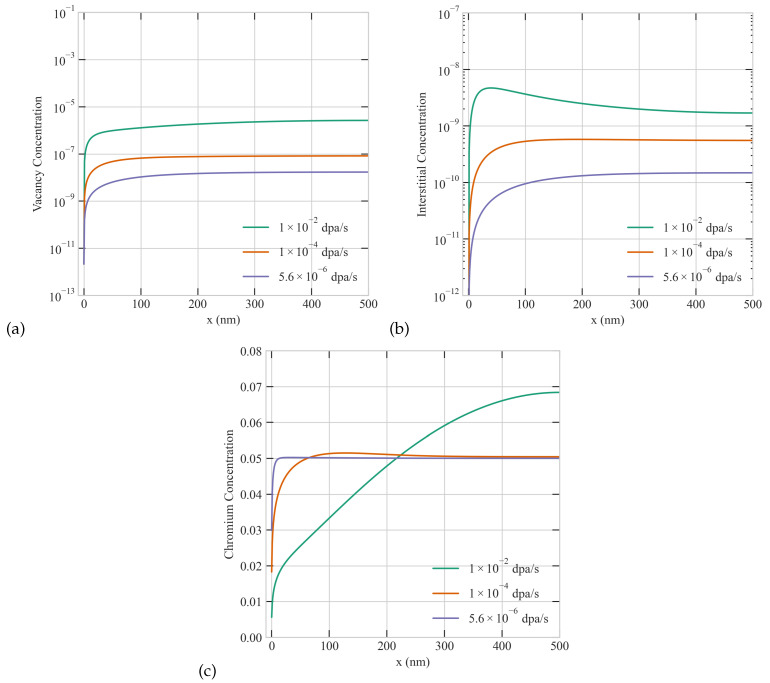
Effect of dose rate on the steady-state concentration profiles of point defects and Cr atoms with 1% production bias: (**a**) vacancy, (**b**) interstitial, and (**c**) Cr concentrations in a 500 nm grain.

Dose rate is an important parameter that reflects the intensity of irradiation. Its value differs depending on the mass and energy of ions. For neutron irradiation, the reactor type, and the location of the component of interest determine the effective dose rate. The dose rate varies by orders of magnitude between different irradiation particles. This renders the attempts of utilizing ion irradiation to emulate neutron irradiation in reactors very challenging. We investigate here the effect of dose rate on RIS in Ni-Cr. The results are presented in Figure 3. To isolate the effect of dose rate, we held constant the size, temperature, production bias, and sink density. As obvious from the figure, radiation damage and RIS are more pronounced at higher dose rate as expected. In particular, the steady state concentrations of both vacancies and interstitials increase with dose rate. Moreover, higher depletion of Cr at the surface/boundary and wider depletion regions are observed at higher dose rates. Furthermore, the higher the dose rate is, the steeper the spatial gradients of the point defects and Cr are. Additionally, the dose rate also influences the surface-mediated patterns and size anomalies discussed earlier. Specifically, the critical size decreases with increasing dose rate. At the lowest dose rate of 5.6 $\times \;10^{-6}$ dpa/s, no accumulation of interstitials by the surface is noticed in a 500 nm grain. Only at dose rates of 1 $\times \;10^{-4}$ dpa/s or higher that those non-classical, non-monotonic patterns start to appear in a 500 nm grain. Stated differently, for each grain size, a critical dose rate can be defined above which non-classical/non-monotonic patterns of point defects develop. Once those patterns are established, RIS becomes more severe. The severity of RIS is manifested through higher/lower concentrations of the segregated/depleted species at the boundary and wider segregation/depletion regions by the boundary as captured in Figure 3c.

### 3.3. The Combined Effect of Dose Rate, Production Bias, and Size on RIS in Ni-Cr

**Figure 4:**
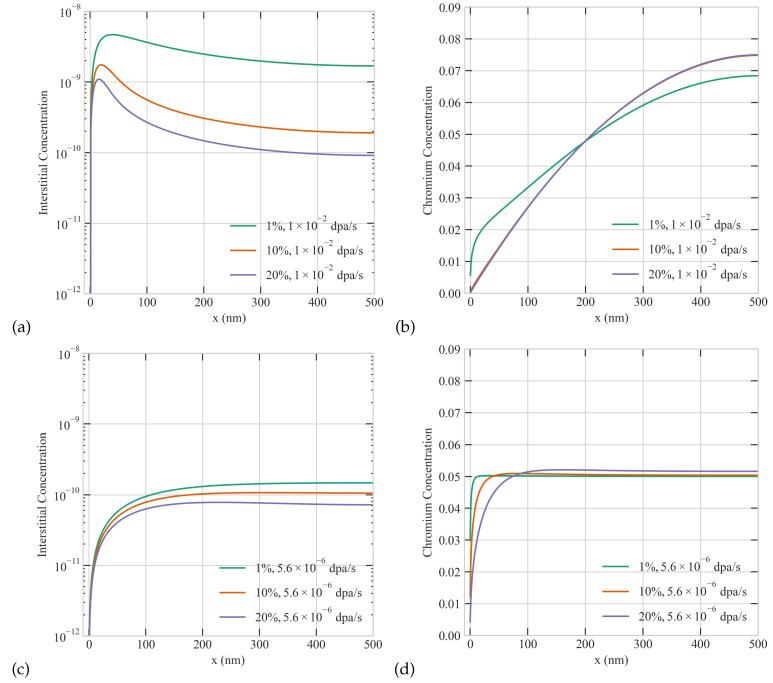
The combined effect of production bias and dose rate on the steady-state concentration profiles of point defects and Cr atoms. Upper row: 1%, 10% and 20% production biases at the dose rate of 1 $\times \;10^{-2}$ dpa/s for (**a**) interstitial (**b**) Cr concentrations. Lower row: 1%, 10% and 20% production biases at the dose rate of 5.6 $\times \;10^{-6}$ dpa/s for (**c**) interstitial (**d**) Cr concentrations.

As evident from the previous sections, production bias and dose rate strongly influence the steady-state concentrations of point defects. Additionally, different types of particle irradiation usually exhibit distinct combinations of those factors. For instance, neutron irradiation in fission reactors will result in high production bias but low dose rate. However, in ion beam accelerators, heavy ion irradiation will lead to high production bias and dose rate, while light ion irradiation produces low production bias with variable dose rate. It is then imperative to study the coupled effect of these factors. To investigate the interplay between dose rate and production bias, we conducted a systematic study considering production bias cases of 1%, 10%, and 20% at dose rates of 1 $\times \;10^{-2}$ and 5.6 $\times \;10^{-6}$ dpa/s in a 500 nm grain. The results are summarized in Figure 4. The depletion of Cr at the grain boundary increases with dose rate and production bias in agreement with the results discussed above. Specifically, we observed that the maximum depletion of Cr at the boundary occurs at the dose rate of 1 $\times \;10^{-2}$ dpa/s with a 20% production bias. Moreover, higher the values of dose rate and production bias lead to the formation of wider depletion regions by the boundary. Based on the trends in Figure 4, it can be concluded that the dose rate influences more the thickness of the depletion layer while the production bias affects more the magnitude of depletion at the grain boundary. Nonetheless, it is their combined effect that eventually quantifies the overall severity of RIS.

**Figure 5:**
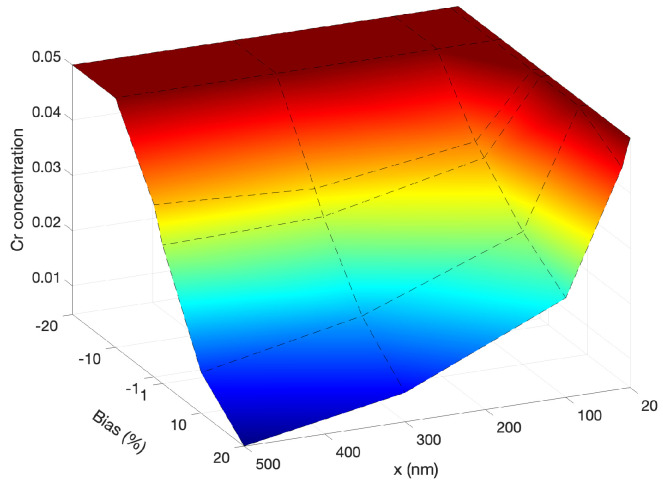
An illustration of the combined effects of size (20, 30, 100, 300, and 500 nm) and production bias (+/−1%, 10% and 20%) on the change of Cr concentration at the grain boundary.

We then turn to the combined effect of production bias and size. Several simulations were performed with production biases between −20% to +20% and sizes from 20 nm to 500 nm. Figure 5 demonstrates the combined effect of size and production bias. The depletion of Cr atoms at the boundary becomes more apparent with size and bias. The concentration of Cr at the boundary reaches its lowest point in a 500 nm grain with a 20% production bias. For the cases with positive production bias, the lowest depletion of Cr is observed in a 20 nm grain with a 1% production bias. For the cases with negative production bias, a similar size dependence appears till −10% bias. However, with higher negative biases (between −10% and −20%), the size dependence vanishes, and no Cr depletion is observed. This is attributed to the preferential exchange of Cr with vacancies which are less present at high negative values of production bias. Our results presented here are consistent with the size-dependent radiation tolerance studies reported before in nano grains [26,27,28]. However, we here capture the transition in radiation tolerance from nano- to micro- size grains while simultaneously accounting for production bias.

### 3.4. Effect of Temperature

**Figure 6:**
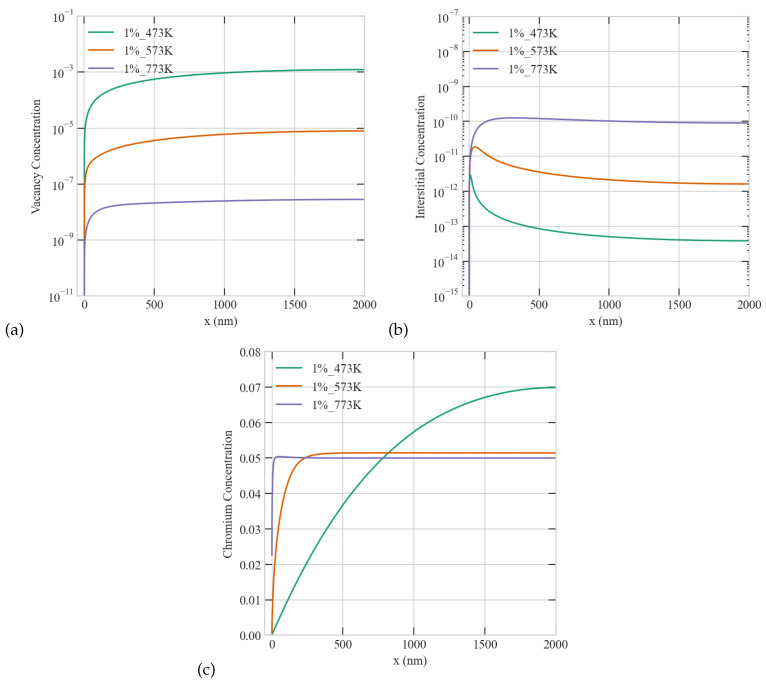
Effect of temperature on the steady-state concentration profiles of point defects and Cr atoms at a dose rate of 5.6 $\times \;10^{-6}$ dpa/s (**a**) vacancy (**b**) interstitial (**c**) Cr concentrations.

The influence of irradiation temperature on the evolution of radiation damage is widely acknowledged, as it affects the mobility of defects and the stability of clusters. Moreover, controlling the irradiation temperature is a widely used approach to enable ion irradiation to emulate neutron irradiation though with limited success. Therefore, understanding the impact of temperature on point defects and atom concentrations is of prominent importance. To that end, systematic simulations were conducted to examine the temperature effect at a fixed dose rate of 5.6 $\times \;10^{-6}$ dpa/s and a 1% production bias. Figure 6 depicts the effect of temperature on point defects in a Ni-Cr grain with 2000 nm radius. As evident from the figure, the interstitials display the surface-mediated patterns discussed before. Moreover, interstitials and vacancies exhibit distinct trends with temperature, i.e., the steady-state concentration of vacancies decreases with temperature while its interstitials counterpart increases with temperature. This is due to the large difference in their migration energies (recall Table 1), which renders vacancies immobile at low temperatures. This consequently influences the dependence of Cr concentration on temperature since vacancies preferably exchange with Cr while interstitials favorably exchange with Ni. The prediction from the results visualized in Figure 6 is that Cr depletion is reduced with temperature, and it is most pronounced at the lowest temperature of 473 K. This prediction, however, contradicts existing experimental data showing that RIS in most alloys, including Ni-Cr, is most apparent at intermediate temperatures [29]. We attribute the deviation between the predictions from the model and experimental data to the dependence of sink density, boundary/surface efficiency, and production bias on temperature. It will be demonstrated in the next sections that one can indeed obtain better agreement with experiments if we account for the temperature dependence of these factors.

### 3.5. Investigating the Interplay between Temperature, Size, and Production Bias on RIS in Ni-Cr

**Figure 7:**
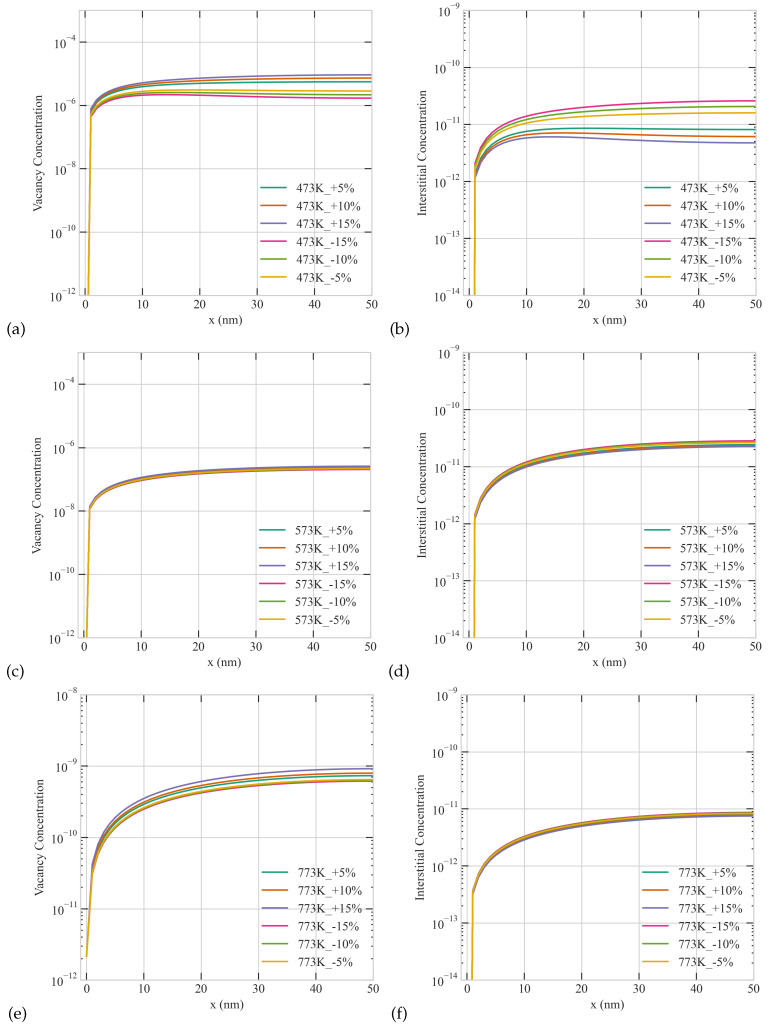
The combined effect of Temperature and production bias on the steady-state concentration profiles of vacancies (**a,c,e**) and interstitials (**b,d,f**) at a dose rate of 5.6 $\times \;10^{-6}$ dpa/s in a 50 nm grain: (**Upper row**) 473 K, (**middle row**) 573 K, (**lower row**) 773 K.

**Figure 8:**
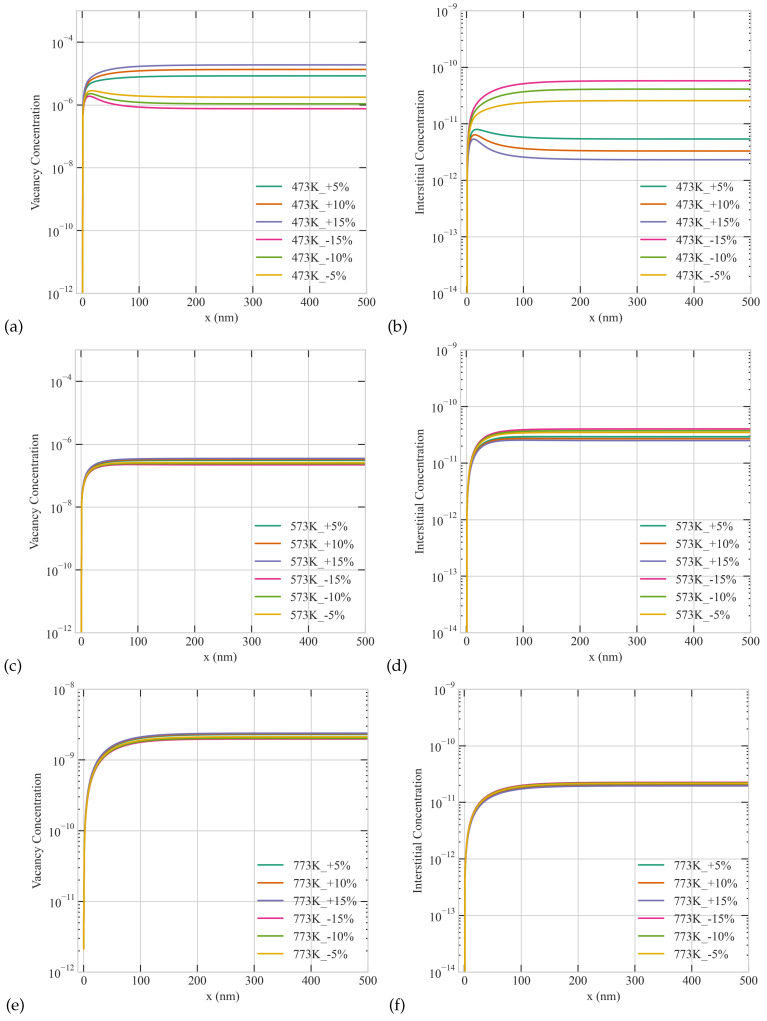
The combined effect of Temperature and production bias on the steady-state concentration profiles of vacancies (**a,c,e**) and interstitials (**b,d,f**) at a dose rate of 5.6 $\times \;10^{-6}$ dpa/s in a 500 nm grain: (**upper row**) 473 K, (**middle row**) 573 K, (**lower row**) 773 K.

**Figure 9:**
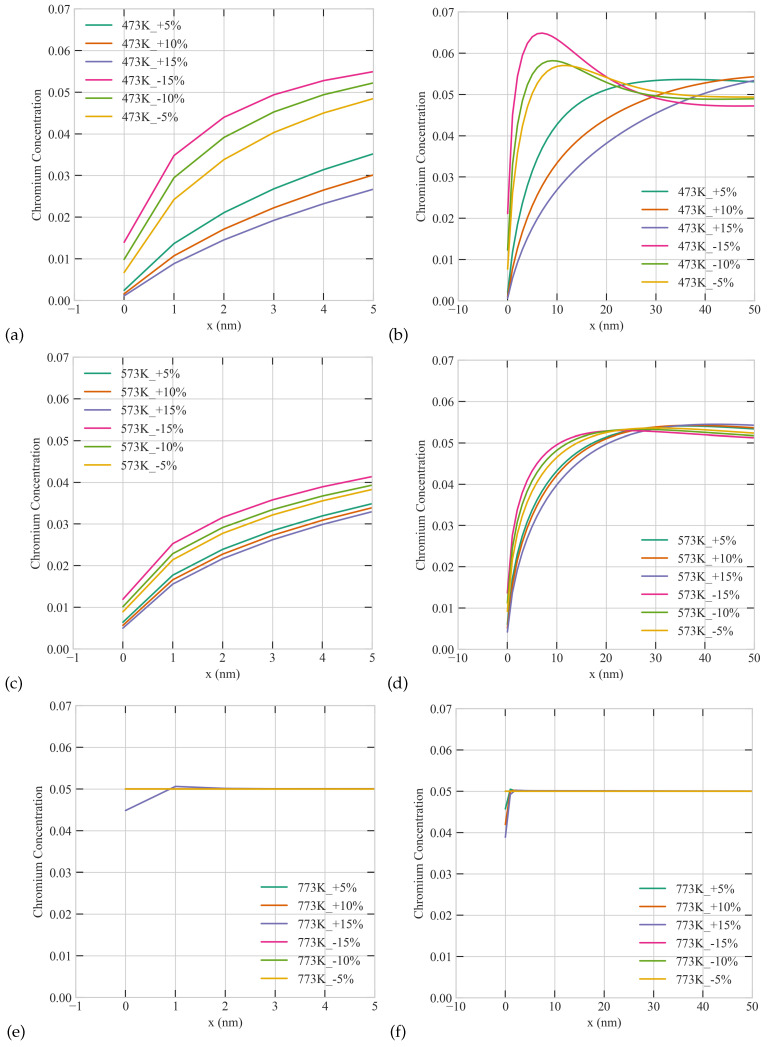
The combined effect of Temperature and production bias on the steady-state concentration profiles of Cr atoms at a dose rate of 5.6 $\times \;10^{-6}$ dpa/s: (**Upper row**) 473 K, (**middle row**) 573 K, (**lower row**) 773 K; left column (**a,c,e**) a close up on the boundary of a 50 nm grain, right column (**b,d,f**) a close up on the boundary of a 500 nm grain.

As we demonstrated above temperature, size, and production bias play crucial roles in the resultant patterns at steady state. In this section, we study the interplay between these factors. To that end, we carried out several simulations with different combinations of these factors. In those simulations, we employed a fixed and uniform sink density of 1 $\times \;10^{22}$ m${}^{-3}$ and a dose rate of 5.6 $\times \;10^{-6}$ dpa/s Two grain sizes of 50 nm and 500 nm, three temperatures 473 K, 573 K, and 773 K, and six production biases +5%, +10%, +15%, −5%, −10%, and −15% were considered, resulting in 36 distinct simulations. The results of these simulations are presented in Figure 7, Figure 8 and Figure 9 below. Figure 7 shows the radiation response of a 50 nm grain as function of temperature and production bias. For the same temperature, a positive production bias results in higher steady state vacancy concentration than a negative bias, and the difference between the two concentrations increases with bias. The situation is reversed for interstitials as expected. However, the effect of bias diminishes with temperature. This is attributed to a change in the dynamics of the system from recombination-dominant to sink-dominant at high temperature and sink density. It is also worthy to note that only monotonic profiles of point defect develop in the small grain under all conditions studied here in agreement with our earlier results. Lastly, the average steady state concentrations of point defects decrease with temperature.

The combined effect of production bias and temperature on the radiation response of the large grain is summarized in Figure 8. Similar to the case of the small grain, positive production bias produces higher vacancy concentrations, and the bias effect reduces with temperature. However, in contrast to the case of the small grain, non-monotonic profiles of point defects develop under subset of conditions. Specifically, at the lowest temperature of 473 K, non-monotonic spatial profiles appear in the steady state concentrations of interstitials/vacancies for positive/negative production bias. At higher temperatures, these non-monotonic patterns are suppressed due to the transition of the kinetics of the system to sink-dominant.

Figure 9 captures the coupled influence of temperature and production bias on the Cr concentrations for the two grain sizes. Cr depletion from the boundaries is more pronounced for the case of positive bias because of the association of Cr migration with vacancies as discussed before. For the same reason, the depletion of Cr decreases with temperature because of the lower vacancy concentrations at higher temperatures. Moreover, non-monotonic patterns also appear in the Cr concentration profiles for the case of the large grain, which can be attributed to their interdependence with vacancies as well. It is worth noting that non-monotonic Cr profiles were observed experimentally in Ni-Cr- and Fe-Cr-based alloys. In particular, the so-called “W” profile reported for austenitic steels in literature [14]. While such profiles were initially considered to be transient and appear only at low dpa, they were subsequently demonstrated to be stable even at higher dpa values [14]. Although the non-monotonic profiles of Figure 9 are distinct from the “W” profile, the results obtained here indicate that such profiles are indeed possible steady-state solutions if the effect of production bias was considered. Note that production bias as the cause of those profiles does not necessarily contradict the possible reasons mentioned in literature before such as the Cr interactions with other solute atoms or the presence of Cr rich phases by the boundary. This is because those reasons mentioned before could affect the clustering and stability of defects and in turn the effective production bias, i.e., those causes are not mutually exclusive.

**Figure 10:**
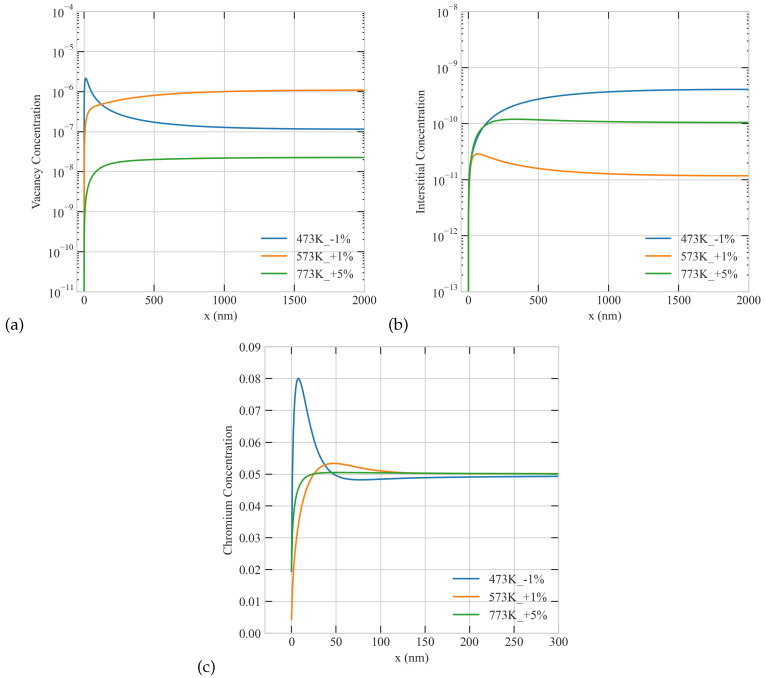
The influence of a temperature-dependent production bias on the steady-state concentration profiles of point defects and Cr atoms for a dose rate of 5.6 $\times \;10^{-6}$ dpa/s in a 2000 nm grain (**a**) vacancy, (**b**) interstitial, and (**c**) Cr concentrations.

For the same value of the production bias, the severity of RIS seems to reduce with temperature (See Figure 6 and Figure 9). As the temperature increases, the concentration of vacancies decreases, and consequently the Cr depletion diminishes. It is, however, often observed that the severity of RIS peaks at intermediate temperatures [29]. This prediction could be reproduced here if one accounts for the temperature dependent production bias. It was indeed shown using molecular dynamics simulations that the stability of the clusters is temperature sensitive and different for vacancies and interstitials [30]. This eventually translates into different numbers of single defects surviving the cascade stage and hence results in an effective production bias. To take that effect into consideration, we ran different simulations with distinct combinations of temperature and production bias. In those simulations, we used a low dose rate of 5.6 $\times \;10^{-6}$ dpa/s and a large grain of 2000 nm to mimic experimental conditions. We assumed, based on [30], that vacancy clusters are more stable than their interstitials counterparts at low temperature and that this trend is reversed as the temperature rises. That trend was attributed in [30] to the overlapping of cascades and the temperature dependence of the annealing of the thermal spike. As evident from Figure 10, when that trend is adopted in our simulations, RIS attends a maximum at the intermediate temperature of 573 K, where Cr concentration at the boundary is the lowest. The non-monotonic profiles of point defects appear at the low and intermediate temperatures but are absent at the highest temperature of 773 K.

### 3.6. Effect of Sink Density

**Figure 11:**
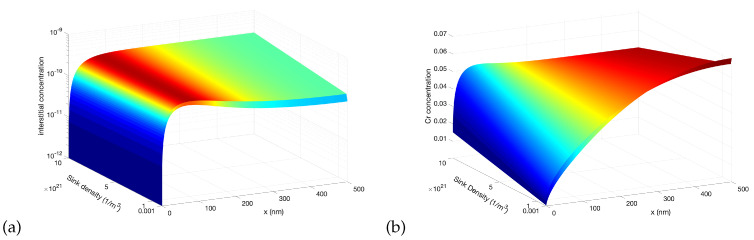
Effect of sink density on the steady-state concentration profiles of point defects and Cr atoms at a dose rate of 1 $\times \;10^{-4}$ dpa/s and 20% production bias: (**a**) interstitials (**b**) Cr concentrations.

The surface-mediated non-monotonic profiles of point defects are more pronounced when the dynamics of the system is recombination-dominant. It is the non-linear reaction term in Equation (1), representing the recombination of point defects, that gives rise to patterning. Similar to temperature, the value of the uniform sink density, which is utilized to approximate the presence of internal sinks such as voids/bubbles and dislocations, is then expected to affect the radiation response of the alloys. To illustrate the influence of sink density on RIS in Ni-Cr, we conducted several simulations with varied sink densities. In all of those simulations, the dose rate was 1 $\times \;10^{-4}$ dpa/s, the production bias was +20%, and the grain size was 500 nm. Figure 11 presents the spatial profiles of the steady-state concentrations of interstitials and Cr as function of sink density. As clear from the figure, the depletion of Cr at the boundary is reduced as the sink density increases. The boundary concentration of Cr is below 0.01 when the sink density is lower than 1 $\times \;10^{21}$ m${}^{-3}$. This is consistent with the earlier observation that RIS becomes more apparent as the dynamics of the system switches from sink-dominant to recombination-dominant.

### 3.7. Effect of the Sink Efficiency of the Surface

We dedicate our last investigation here to study the effect of the boundary/surface efficiency of RIS. The sink efficiency refers to the capability of a grain boundary or a surface in general to absorb point defects. As was demonstrated from both experiments and atomistic simulations, the value of this parameter depends on the structure of the interface. Incoherent interfaces are expected to have higher efficiency than coherent interfaces because of the lower density and order of their atomic structure, which facilitates the accommodation and recombination of more point defects. A boundary/surface is said to be perfect if it has 100% efficiency, i.e., its capacity to absorb point defects is unlimited. On the other hand, an imperfect surface is a surface with a limited capacity. For a perfect surface, the concentrations of the point defects there are always the equilibrium concentration for a given temperature, e.g.,

(6) $$\begin{array}{cc} \; & C_{v}{|}_{0}=C_{v}^{e}\\ \; & C_{i}{|}_{0}=C_{i}^{e} \end{array}$$

The concentrations of point defects at imperfect interfaces, however, are always higher than their equilibrium values because of lower fluxes/flow rates to these boundaries due to the saturation of their absorption capacity. To account for this effect in our model, the sink efficiency κ is defined as the ratio between the defect flux into an imperfect boundary $\left(J_{imperfect}\right)$ and the defect flux into a perfect boundary $\left(J_{perfect}\right)$ to describe the ability of a grain boundary to absorb point defects.

(7) $$\begin{array}{cc} \; & \kappa =\frac{J_{imperfect}}{J_{perfect}} \end{array}$$

To simulate an imperfect boundary, we first solve the model for perfect boundary conditions, employing the equilibrium boundary conditions to obtain the corresponding defect flux, $\left(J_{perfect}\right)$. Subsequently, for imperfect boundary scenarios, we assign a fraction of that perfect flux as the new boundary condition:

(8) $$\begin{array}{cc} \; & J_{imperfect}=\kappa J_{perfect} \end{array}$$

**Figure 12:**
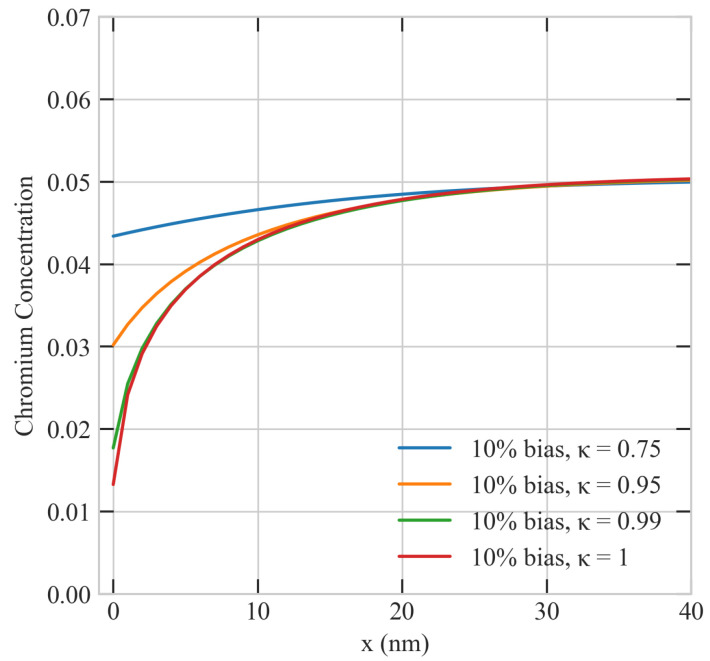
Effect of the sink efficiency of the surface on the concentration profiles of Cr atoms. κ is the sink efficiency, with κ = 1 representing a perfect boundary and κ < 1 representing imperfect boundaries.

To represent boundaries with variable efficiency, we ran several simulations with different values of κ. In those simulations, we employed a grain size of 72,500 nm, a uniform sink density of 1 $\times \;10^{20}$ m${}^{-3}$, a dose rate of 5.6 $\times \;10^{-6}$ dpa/s, a production bias of +10%, and a temperature of 773 K. The results of these simulations are summarized in Figure 12. It is obvious from the figure that the sink efficiency (κ) strongly influences the steady-state spatial profiles of Cr. The depletion of Cr atoms is reduced as the surface efficiency decreases. The perfect surface (κ = 1) has the lowest surface concentration of Cr and the steepest Cr gradient. This is consistent with the establishment of higher flow rates of point defects moving to the surface as its character changes from imperfect to perfect (as κ increases).

### 3.8. Comparison with Experiments

**Figure 13:**
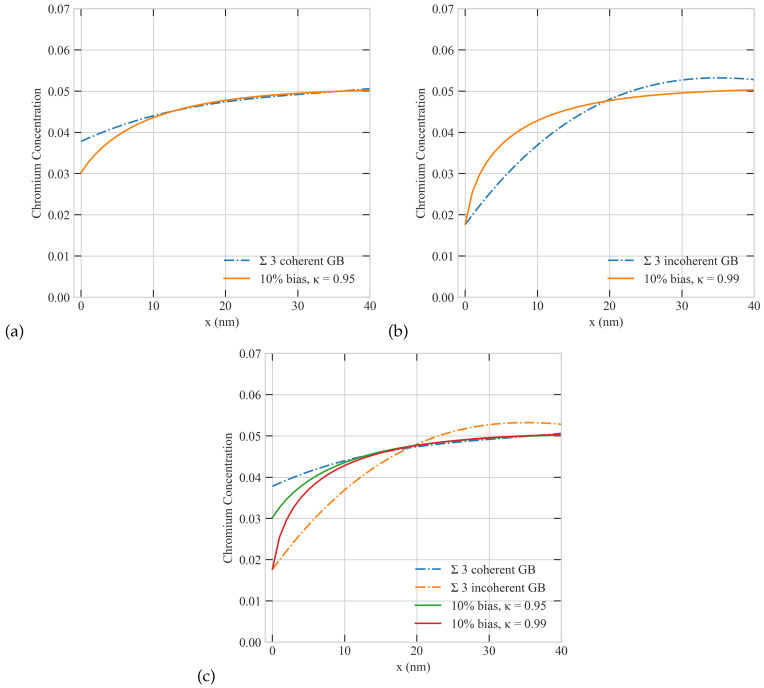
Comparisons of model predictions with data from RIS experiments [12] (Solid lines represent the predictions of the model and dotted/dashed lines are the experimental data) (**a**) coherent grain boundary (GB), (**b**) incoherent GB, (**c**) an incoherent GB has a higher sink efficiency (κ) than a coherent GB.

In the previous sections we presented an elaborate investigation of RIS in Ni-Cr alloys. The influences of the principal factors affecting RIS were studied and analyzed. We related our model predictions qualitatively to experimental data and theoretical estimates. We now attempt to quantitatively validate the results from our simulations against related data from RIS experiments [12]. To facilitate a meaningful comparison, we simulated RIS in Ni-5Cr under similar conditions to those reported in [12]. We utilized a domain size (e.g., grain radius) of 72,500 nm, a sink density of 1 $\times \;10^{20}$ m${}^{-3}$, a dose rate of 5.6 $\times \;10^{-6}$ dpa/s, and a temperature of 773 K. We employed an intermediate production bias of +10% to mimic heavy ion irradiation at 773 K. Figure 13 shows the comparison the predictions of the model and the corresponding experimental data. Generally speaking, the figure demonstrates a good agreement between the model’s predictions and the results from experiments. They both show lower concentration of Cr by the incoherent (near perfect) boundary than its coherent counterpart. The deviations between the predictions and data for Cr are less than 0.01, which is probably within the accuracy limit of the experimental measurements. The spatial gradients are also well reproduced in our simulations, particularly for the coherent boundary. The differences in the spatial gradients for the incoherent boundary might stem from secondary factors that were neglected in the current study. For instance, the distribution of the uniformly distributed sinks, their spatial profiles, or the spatial dependence of the production bias. Taking into account these factors will be considered in future studies.

## 4. Concluding Remarks

In this manuscript, we performed an extensive investigation of the RIS phenomenon in Ni-Cr alloys, exploring the main factors influencing RIS. Specifically, the effects of the defect absorption efficiency of the surface, grain size, production bias, dose rate, temperature, and sink density on the RIS were thoroughly analyzed. We conducted simulations that test the influence of those factors separately and in different combinations to advance the current understanding of RIS. Moreover, we compared the predictions of our simulations with experimental data whenever possible.

Production bias was shown to strongly influence the interactions between point defects and interfaces/surfaces and hence plays a major role in the process of RIS in alloys. This is because production bias compromises the neutrality of these interactions, leading to the preferential absorption of one type of point defects by the boundary. In contrast to a situation with zero bias, the presence of any bias indicates that, for a system to attain a steady state, the surface or boundary must absorb more of the defect species produced at a faster rate. This means the surface must favor the absorption of the species generated more rapidly and disfavor the one produced more slowly. Thus, in systems with a non-zero production bias, truly neutral surfaces cannot exist. The species that the surface or boundary less efficiently absorbs tends to accumulate near that boundary. This eventually leads to the establishment of distinct surface-mediated patterns of point defects. These spatial patterns of vacancies and interstitials, in turn, results in the development of non-monotonic spatial profiles of solute atoms and the formation of heterogeneous distribution of extended defects in the vicinity of the surfaces/interfaces, as was observed experimentally [14,24,25]. Positive production bias, when vacancies are produced with an effectively higher rate than interstitials, drives more Cr depletion at the grain boundary. This is attributed to the preferential exchange of Cr atoms with vacancies, which are more abundant in such scenario. Moreover, it was predicted that a temperature-dependent production bias, changing from negative at low temperatures to positive at higher temperatures, was necessary to reproduce the correct temperature-dependence of RIS in alloys, where RIS is often reported to peak at intermediate temperatures.

The extent of radiation damage and severity of RIS increase with production bias, dose rate, and grain size. A critical value for each of those factors can be defined at which non-monotonic steady-state spatial profiles of point defects and Cr atoms start to appear. On the other hand, at high temperatures and/or sink density, radiation damage and RIS severity are suppressed. This is associated with a transition in the dynamics of the system from recombination dominant to sink dominant, at which point the loss of point defects to sinks becomes high enough to reduce permanent radiation damage.

The model predictions were validated against the experimental data of [12]. It was shown that both production bias and reduced surface’s sink efficiency are required to reproduce the experimental results. The model’s forecasts consistently agreed with the data, with differences in the Cr atom fraction at the boundary of less than 0.01, which is likely within the accuracy limit of the experimental measurements. For a coherent grain boundary, an almost exact match was demonstrated between the steady-state spatial profiles from the experiments and simulations. However, for the case of incoherent grain boundary, minor variations in the profiles persisted. We believe those deviations stem from the spatial dependencies of production bias and sink density which were unaccounted for in this study. Investigating the effects of these factors on the RIS process in alloys will be pursued in future studies.

## Figures and Tables

**Table 1:** RIS model input parameters [12].

Parameter	Value
Pre-exponential factor for Ni interstitial diffusivity	5.04 $\times \;10^{-8}$ m${}^{2}$/s
Pre-exponential factor for Cr interstitial diffusivity	3.20 $\times \;10^{-7}$ m${}^{2}$/s
Pre-exponential factor for Ni vacancy diffusivity	1.85 $\times \;10^{-4}$ m${}^{2}$/s
Pre-exponential factor for Cr vacancy diffusivity	2.26 $\times \;10^{-4}$ m${}^{2}$/s
Activation energy for Ni interstitial diffusivity	0.30 eV
Activation energy for Cr interstitial diffusivity	0.37 eV
Activation energy for Ni vacancy diffusivity	1.16 eV
Activation energy for Cr vacancy diffusivity	1.10 eV
Vacancy formation energy	1.79 eV
Interstitial formation energy	4.0 eV

## Data Availability

The data presented in this study are available upon request from the corresponding author.
